# Supplementary material for: Two Novel Betarhabdovirins Infecting Ornamental Plants and the Peculiar Intracellular Behavior of the Cytorhabdovirus in the Liana Aristolochia gibertii
Source: Viruses. 2024 Feb 21;16(3):322. doi: 10.3390/v16030322 (PMC10976027; doi:10.3390/v16030322)
Supplement: Supplementary file 1 [file viruses-16-00322-s001.zip › viruses-2855927-supplementary.pdf]

Supplementary Table S1. List of primers used in the study of the betarhabdoviruses AaCV and FaJV.

Primer	Sequence 5′-3′	Target position of the specific primers in the virus antigenomic strand	Reference
**Degenerate primers for the detection of plant rhabdoviruses**			
RhabF	GGATMTGGGGBCATCC		[14]
RhabR	GTCCABCCYTTTTGYC		
**Specific primers for the detection of AaCV**			
AaCV 1F	TTGGAGCACATTATACATTAATC	67-89	
AaCV 1Fa	AAGAGAGTGCAAGGATTTG	170-188	
AaCV 1R	TCTTTCAGTGCAGCATTATG	1040-1059	
AaCV 1Ra	TTCCTAGTCTTTCTGGTACTC	420-440	
AaCV 2F	CAATTTGGTTGCTCAATAGTC	1005-1025	
AaCV 2R	CAGACCTTCCTCTCTTGAG	1882-1900	
AaCV 3F	GCTGGAAAGAAAGGAGAAG	1812-1830	
AaCV 3R	TGCCTTATCTTGCTGTTTTG	2608-2027	
AaCV 4F	GGACCCAGAGGTGATAAAAG	2528-2547	
AaCV 4R	GACTGATCCTACGTCGTTC	3316-3334	
AaCV 5F	CGATTAAATTTCAGGAGATACAG	3246-3268	
AaCV 5R	CTTTCTCATCATGTTTGAACAG	4093-4114	
AaCV 6F	ATCATGTTACGCCATTGAC	4040-4056	
AaCV 6R	AAACCTTCCAAAGAGCAAG	4970-4988	
AaCV 7F	GATTTGCGACTCGAAGATAG	4914-4933	
AaCV 7R	GCTTTACCAACTTACAGTGATG	5857-5878	
AaCV 8F	ATGATTGCTGCAAAAGGAG	5812-5830	
AaCV 8R	AAGATGGAAGTCCCCTATG	6803-6821	[This study]
AaCV 9F	TCTTTCTGATCTTGATTTCGAG	6755-6776	
AaCV 9R	TCCTCGACATCAGAAACTG	7684-7702	
AaCV 10F	AATCTGAAAGTGTCTGGATTTG	7632-7653	
AaCV 10R	TTAAAAAGGTGAAAGGTAGTCTC	8589-8611	
AaCV 11F	CTGCATAAATATGGATTTCGAAAAG	8543-8567	
AaCV 11R	TATCTTGGCACAGTATTGAATAG	9603-9625	
AaCV 12F	AAGGGTTTAGAGAAGAAAGGA	9538-9558	
AaCV 12R	TTATACTTTTGTTCTGCATTGC	10453-10474	
AaCV 13F	CAACAATCTAGCGTCCATTAC	10385-10405	
AaCV 13R	GCAAAACCATCTCTGATCTC	11212-11231	
AaCV 14F	TTAAAGGGATGTTGAGTATGTTC	11161-11183	
AaCV 14R	CATTGGATAAATTCAGTATGTTTTC	12054-12078	
AaCV 15F	AGCATACCCAACACTGAAC	12000-12018	
AaCV 15Fa	CAACTCTCTCTGCGATTATTAG	12747-12768	
AaCV 15R	ATAAATGTATGATGACATAGTTTTACTG	13073-13100	
AaCV RACE-5′	GGGTGGTTCTATGAAAGCGGGATAACACAATGG	12600-12632	
AaCV RACE-3′	TCTCCAAACGGATTTGCTGGAGCCTTCG	552-579	
**Specific primers for the detection of** FaJV			
FaJV_N_F	GAGCGGTTGTGAGTTAGCAG	1039-1058	[This study]
FaJV_N_R	TCGGTGTGTATCTTGCCTGT	1409-1428	
